# First clinical experience with fractionated intracavitary radioimmunotherapy using [^177^Lu]Lu-6A10-Fab fragments in patients with glioblastoma: a pilot study

**DOI:** 10.1186/s13550-023-01029-7

**Published:** 2023-09-04

**Authors:** Wolfgang Roll, Michael Müther, Guido Böning, Astrid Delker, Nils Warneke, Franz-Josef Gildehaus, Michael Schäfers, Walter Stummer, Reinhard Zeidler, Hans-Jürgen Reulen, Lars Stegger

**Affiliations:** 1 Department of Nuclear Medicine, University Hospital Münster, Albert-Schweitzer-Campus 1, 48149 Münster, Germany; 2 West German Cancer Centre, Münster, Germany; 3 Department of Neurosurgery, University Hospital Münster, Münster, Germany; 4 Department of Nuclear Medicine, University Hospital, Ludwig-Maximilians-University Munich, Munich, Germany; 5 European Institute for Molecular Imaging, University of Münster, Münster, Germany; 6 Department of Otorhinolaryngology, University Hospital, Ludwig-Maximilians-University Munich, Munich, Germany; 7 Institute of Structural Biology, Helmholtz Center Munich, Munich, Germany; 8 Department of Neurosurgery, University Hospital, Ludwig-Maximilians-University Munich, Munich, Germany

**Keywords:** Radioimmunotherapy, Carbonic anhydrase XII, Immunoglobulin Fab fragments, Glioblastoma, Lutetium-177

## Abstract

**Background:**

Following resection and standard adjuvant radio- and chemotherapy, approved maintenance therapies for glioblastoma are lacking. Intracavitary radioimmunotherapy (iRIT) with ^177^Lu-labeled 6A10-Fab fragments targeting tumor-associated carbonic anhydrase XII and injected into the resection cavity offers a novel and promising strategy for improved tumor control.

**Methods:**

Three glioblastoma patients underwent tumor resection followed by standard radio- and chemotherapy. These patients with stable disease following completion of standard therapy underwent iRIT on compassionate grounds. After surgical implantation of a subcutaneous injection reservoir with a catheter into the resection cavity, a leakage test with [^99m^Tc]Tc-DTPA was performed to rule out leakage into other cerebral compartments. IRIT comprised three consecutive applications over three months for each patient, with 25%, 50%, 25% of the total activity injected. A dosimetry protocol was included with blood sampling and SPECT/CT of the abdomen to calculate doses for the bone marrow and kidneys as potential organs at risk.

**Results:**

All three patients presented without relevant leakage after application of [^99m^Tc]Tc-DTPA. Two patients underwent three full cycles of iRIT (592 MBq and 1228 MBq total activity). One patient showed histologically proven tumor progression after the second cycle (526 MBq total activity). No relevant therapy-associated toxicities or adverse events were observed. Dosimetry did not reveal absorbed doses above upper dose limits for organs at risk.

**Conclusions:**

In first individual cases, iRIT with [^177^Lu]Lu-6A10-Fab appears to be feasible and safe, without therapy-related side effects. A confirmatory multicenter phase-I-trial was recently opened and is currently recruiting.

**Supplementary Information:**

The online version contains supplementary material available at 10.1186/s13550-023-01029-7.

## Background

Despite continuous efforts in basic science and clinical research, glioblastoma (GBM) is still associated with only poor prognosis. After surgical cytoreduction and standard adjuvant treatment with radiation and chemotherapy, approved maintenance therapies are lacking. In line with the infiltrative glioma biology, most disease recurrences are observed in close adjacency to the resection cavity [1]. Previously, local radioactively labelled therapies for treatment of GBM [2, 3] have been reported. Different tumor microenvironment targets were addressed with antibodies or antibody fragments labelled with ß-emitting isotopes, namely iodine-131, yttrium-90 or lutetium-177 [2, 4–6]. Bypassing the brain-blood barrier, instillation of radiopharmaceuticals into the resection cavity allows for higher tumor-absorbed doses when compared to a solely intravenous application. Intracavitary radioimmunotherapy (iRIT) with an adaption of activity according to the resection cavity volume allows for a patient-specific treatment. Previous studies on iRIT with ^90^Y- and ^131^I-labelled antibodies targeting tenascin C have shown prolonged survival of high-grade glioma patients [2].

Carbonic anhydrase XII (CA-XII) is a cell surface glycoprotein overexpressed on glioma cells but not in healthy brain parenchyma [7]. Evaluation of a ^177^Lu-labelled antibody fragment, 6A10-Fab, showed promising results in preclinical in vitro and in vivo animal studies with high specific binding [4]. The use of smaller antibody fragments instead of a full antibody construct is associated with improved tissue penetration [4]. Finally, the theranostic radionuclide lutetium-177 allows for imaging, dosimetry and treatment at the same time.

The aim of this work is to report first clinical experiences with iRIT using ^177^Lu-labeled 6A10-Fab, targeting tumor-associated CA-XII.

## Materials and methods

### Patients

**Fig. 1:**
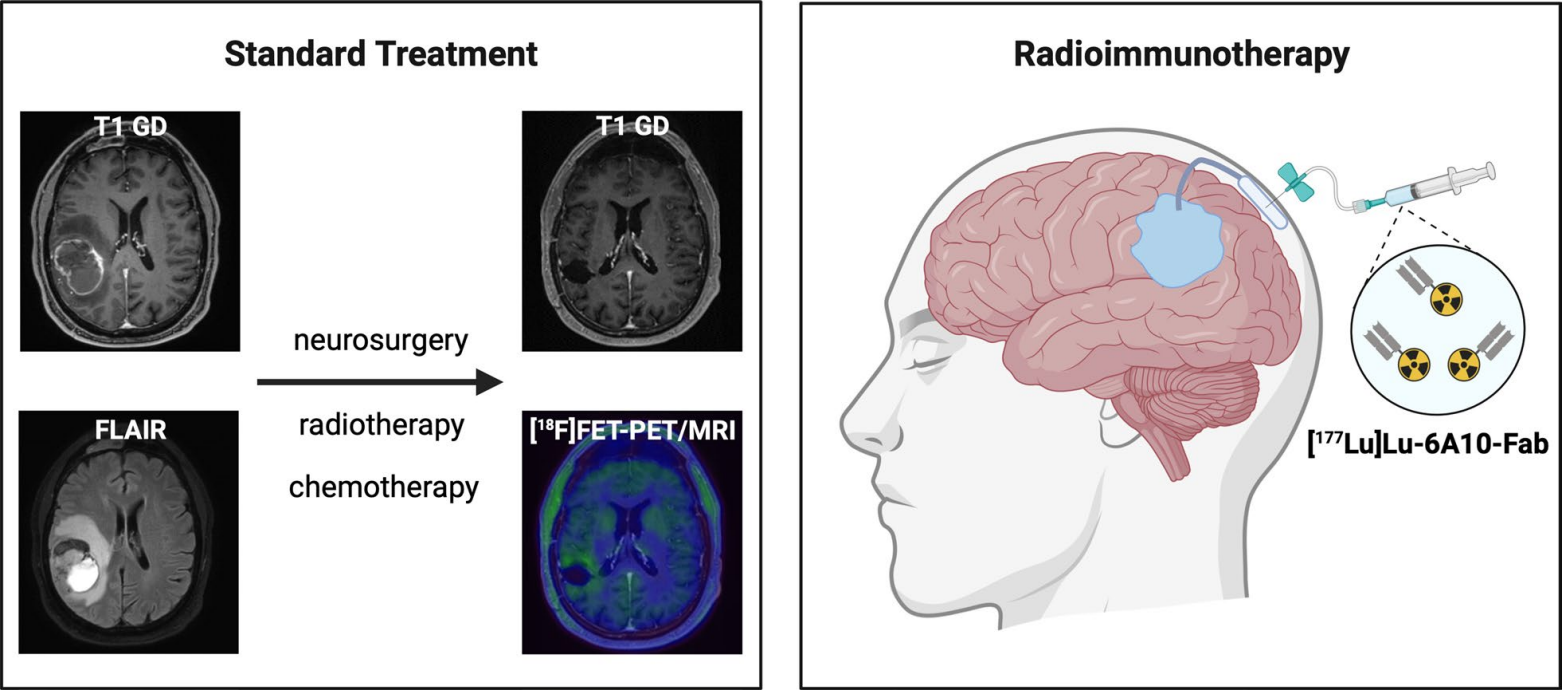
Radioimmunotherapy is applied after standard treatment of glioblastoma. Standard treatment includes surgical resection followed by radiotherapy and chemotherapy. Exemplary MRI, T1 post contrast and FLAIR of a right temporal glioblastoma, without contrast enhancing or [^18^F]FET-PET positive tumor

Three patients underwent microsurgical resection of glioblastoma. After completion of concurrent radiochemotherapy and adjuvant chemotherapy, the patients presented with stable disease without relevant residual tumor on PET/MRI using the radiotracer [^18^F]Fluoroethyl-L-tyrosine ([^18^F]FET) (Fig. 1). This was defined as absence of tumor suspicious [^18^F]FET-uptake (TBRmax > 1.8). Residual contrast enhancement in a tissue volume below 1cm^3^ in MRI was tolerated. Based on a tumor board decision, fractionated iRIT with [^177^Lu]Lu-6A10-Fab was offered on a compassionate use basis for maintenance therapy. All three patients gave informed consent and were treated before initiation of a prospective multicenter phase I trial (NCT05533242). All three patients were treated according to the study protocol with the exception of a different sequencing of activities (see below) without meeting the inclusion criteria because of slightly too small or too large resection cavities (*n* = 2) and contrast enhancement adjacent to the cavity (*n* = 1). Retrospective data analysis and publication of the three cases was approved by the local ethics committee (Ref: 2023-411-f-S; Ethik-Kommission Westfalen Lippe).

### PET/MRI

Patients underwent dynamic [^18^F]FET-PET/MRI on a Siemens Biograph mMR 3-T PET/MRI system (Siemens Healthineers, Erlangen, Germany) before and 4–6 weeks after the last application of fractionated iRIT. [^18^F]FET-PET was performed following the institutional standard protocol that is based on national and international recommendations. [^18^F]FET PET was performed with “listmode” acquisition, where data from each detection event are stored individually for retrospective data sorting and reconstruction. Data were acquired for 40 min starting with the application of 2.5 MBq/kg bodyweight of [^18^F]FET and retrospectively sorted into 14 frames: 1–5: 1 min, 6–10: 3 min, 11–14: 5 min. Additionally, 20–40 min summation images were reconstructed for uptake quantification. The structural MRI protocol included 3D T1-weighted image stacks (MPRAGE) pre- and post-contrast agent injection (0.1–0.2 ml/kg Gadovist 1 mmol/ml; Bayer, Leverkusen, Germany) using a power injector. Additionally, 3D T2-weighted FLAIR, DTI, SWI and a T2*-weighted EPI image sets were obtained.

### iRIT

All patients underwent surgical implantation of an injection reservoir (Codman Holter Rickham Reservoir 9.5 mm, Integra LifeSciences, Princeton, NJ, USA) connected to a central catheter (Codman Bactiseal, Integra LifeSciences, Princeton, NJ, USA) accessing the resection cavity (Fig. 1). Before initiation of iRIT all patients underwent a test injection, using the implanted injection reservoir, to rule out unintended leakage into the subgaleal, epidural, subdural or subarachnoid spaces. A median dose of 108 MBq [^99m^Tc]Tc-DTPA in 1–1.5 ml fluid was administered after a similar amount of cerebrospinal fluid was withdrawn. Planar whole-body scintigraphy and SPECT/CT of the head were performed 30–60 min and 4 h after application following institutional procedures adapted from [^99m^Tc]Tc-DTPA cerebrospinal fluid scintigraphy. After negative leakage testing, three consecutive courses of [^177^Lu]Lu-6A10-Fab were scheduled over three months. In a sterile fashion, the injection reservoir was accessed with a 21-gauge needle for injection of 1.2–3 ml of [^177^Lu]Lu-6A10-Fab directly after withdrawal of a similar volume of cerebrospinal fluid. The injected activity was adapted to the resection cavity volume, based on previously reported diffusion properties, pharmacokinetics and dosimetry evaluations of larger iRIT patient cohorts [4, 8]. The volume of the resection cavity was assessed on contrast-enhanced 3D T1-weighted MRI images using a semiautomatic contouring approach provided by the Syngo.via software (Siemens Healthineers, Erlangen, Germany).

The total activity calculated according to the aforementioned study protocol was administered in a fractionated fashion, with three doses given with four-week intervals. For safety reasons in first-in-man application, we started with 25% of the total activity followed by 50% and 25% instead of the 50–25–25% sequencing defined in the study protocol. After application of [^177^Lu]Lu-6A10 Fab patients were isolated on a nuclear medicine therapy ward until whole-body retention, as measured by the local radiation dose rate, reached the level legally required for patient discharge in Germany. Patients received brain edema prophylaxis with 3 × 4 mg/day dexamethasone for 4 days, starting 24 h before injection.

### [^177^Lu]Lu-6A10 Fab production

GMP Fab-CHX-A’’-DTPA fragment was produced by BIBITEC GmbH & Co. KG (Germany),no-carrier-added Lutetium-177 was been provided by ITM MI GmbH (Germany). Radiolabeling and GMP manufacturing of Lu-177-Fab fragment was performed at Seibersdorf Laboratories GmbH (Austria). The labelling process has already been described in a previous publication by Fiedler et al. [4]. In brief, the radiolabeling reaction has been performed in an acetate buffer solution by incubation of the Fab fragment and Lutetium-177 preparations at room temperature, followed by purification of the reaction mixture on PD-10 single-use column (Merck Germany). Final formulation has been adjusted with the sterile saline solution for injection. Radiochemical and chemical purity as well as identity of the final product has been monitored.

### Dosimetry and TOXICITY

Dosimetry was performed as previously reported [9]. In brief: To assess abdominal organs, we used planar whole-body scintigraphy and SPECT/CT (Symbia T2; Siemens Healthineers, Erlangen, Germany) of the abdomen, approx. 2 h, 24 h, 48 h, 72 h and at a late time point 5–7 days after injection (Additional file 1: Figure S1). Furthermore, multiple blood samples were taken at the day of activity administration and at the time of scanning. Quantitative SPECT/CT image data were reconstructed as previously described [9]. To estimate absorbed kidney doses of kidneys and hematopoetic bone marrow, time-integrated activities were derived from a linear interpolation of available quantitative SPECT/CT and blood sample measurements and by assuming physical decay thereafter. Appropriate dose conversion factors were retrieved from the opendose.org website. SPECT/CT of the brain was performed at the same time points to exclude leakage.

Standard blood work up was done before iRIT, at each treatment cycle and during follow-up. Assessment of toxicity was conducted following the Common Terminology Criteria for Adverse Events (CTCAE v5.0, https://evs.nci.nih.gov/ftp1/CTCAE/).

## Results

All patients underwent iRIT with [^177^Lu]Lu-6A10-Fab between 5/2021 and 7/2022. Patients 1 and 3 underwent all three cycles, whereas patient 2 underwent two cycles only.

### Patient 1

**Fig. 2:**
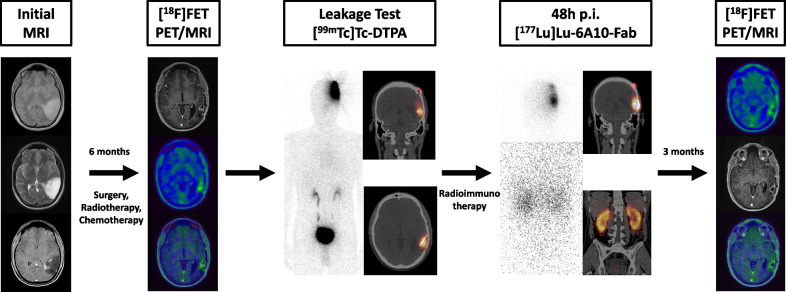
Patient 1 underwent gross total resection and radiochemotherapy. [^18^F]FET-PET/MRI did not show relevant residual tumor tissue. No relevant leakage was detected. Fourty-eight hours after application of the first cycle of iRIT, whole body imaging showed uptake in the resection cavity and in the kidneys

A 41-year-old male, with transient motor aphasia at initial diagnosis, was diagnosed with a left parietal GBM (IDH-mutated, MGMT-methylated) in 2020, according to the 2016 WHO classification update. After gross total resection of the tumor the patient underwent standard radio- and chemotherapy. [^18^F]FET-PET/MR was performed six month after initial resection and after radiochemotherapy. The volume of the resection cavity was measured as 4.5 ml. The leakage test did not reveal any relevant leakage (Fig. 2) into other cerebral structures. The patient underwent three courses of iRIT (cumulative activity: 592 MBq) without adverse events during or after application. 22 months after iRIT, at 38 months after initial diagnosis, the patient remains stable.

### Patient 2

**Fig. 3:**
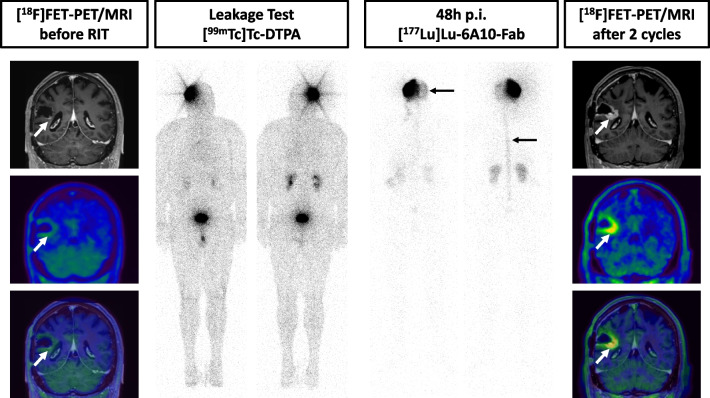
Pretherapeutic [^18^F]FET-PET/MRI of patient 2 revealed a contrast enhancement adjacent to the resection cavity (arrow) without a relevant [^18^F]FET hot spot. Whole body images after [^99m^Tc]Tc-DTPA injection did not reveal relevant leakage. During the second cycle post therapeutic images 48 h p.i. showed leakage into the subarachnoid space (arrows). After two cycles of RIT, [^18^F]FET-PET/MRI marks suspicion of tumor progression (arrow)

In a 56-year-old male, with left hemiparesis at initial diagnosis in 2021, imaging and histopathology revealed the diagnosis of a right parietal GBM (IDH-wildtype, MGMT-not-methylated). Following standard treatment with resection, radio- and chemotherapy, [^18^F]FET-PET/MRI after standard therapy revealed contrast enhancement adjacent to the resection cavity without a relevant [^18^F]FET hot spot. The decision was made to take a biopsy from this region. Histology revealed therapy-associated changes and no residual or recurrent tumor and an injection reservoir for iRIT was implanted accordingly. The postoperative resection cavity volume was 7 ml. Initial [^99m^Tc]Tc-DTPA leakage testing was negative. This patient received two cycles of iRIT (cumulative activity: 526 MBq). During the second cycle an asymptomatic leakage into the subarachnoid space was noted (Fig. 3, Additional file 1: Figure S1). Three weeks after the second cycle the patient presented with seizures. Imaging revealed progression of contrast enhancing tissue adjacent to the resection cavity with a hotspot in [^18^F]FET-PET/MRI. After subsequent microsurgical resection, neuropathology found GBM cells resulting in the diagnosis of progressive disease. No further iRIT was performed, and the patient underwent re-irradiation and chemotherapy. (Fig. 3). The patient died six months after iRIT.

### Patient 3

**Fig. 4:**
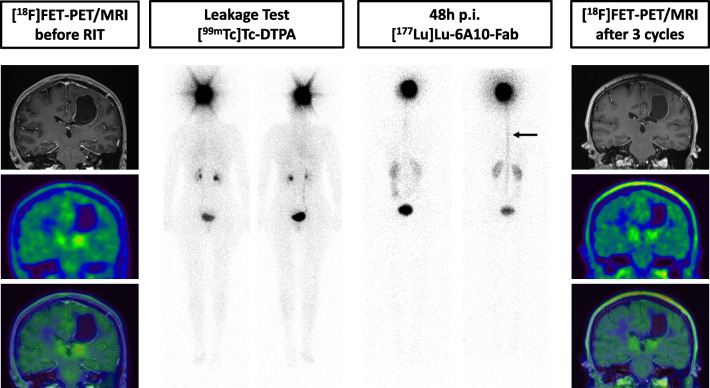
^[18^F]FET-PET/MRI of patient 3 did not reveal relevant residual disease. Leakage test with [^99m^Tc]Tc-DTPA was negative. During the second cycle, low level leakage into the spinal canal was detected (arrow). [^18^F]FET-PET/MRI after iRIT showed stable disease

A 38-year-old female presented with a cystic central lobule GBM (IDH-wildtype, MGMT-methylated). At initial diagnosis, the patient presented with paresthesias of the right foot and of the right arm. For functional reasons, a complete resection was not possible, and an injection reservoir was left for easy access to the space-occupying tumor cyst. Tapping the reservoir was not necessary during adjuvant radio- and chemotherapy. Accordingly, in this patient, additional surgery for reservoir implantation was not necessary. The cavity measured 27 ml. Leakage testing was negative (Fig. 4). The patient received three cycles of iRIT (cumulative activity: 1288 MBq). During the second cycle, a low level of asymptomatic leakage into the spinal canal was detected. [^18^F]FET-PET/MRI four weeks after iRIT showed stable disease without visible signs of tumor recurrence. The patient remains stable 10 months after iRIT and 22 months after initial diagnosis.

### Toxicity and Dosimetry

There were no extracerebral adverse effects in bone marrow and kidneys attributable to iRIT in any of the subjects (Table 1). Patient 1 presented with alterations in blood levels of leukocytes, haemoglobin, and platelets already before RIT, no further degradation was observed after iRIT. Patient 3 showed slightly low but constant platelet counts during iRIT already present at baseline. Dosimetry did not reveal absorbed dose estimates above the upper dose limits for organs at risk. In all three patients, for the kidneys, the estimated absorbed dose was approximately 2.1 ± 1.4 mGy/MBq and 0.03 ± 0.01 mGy/MBq for the hematopoietic bone marrow.

**Table 1:** Side effects of intracavitary Radioimmunotherapy according to CTCAE criteria (version 5.0)

	Patient 1			Patient 2			Patient 3		
	Baseline	After one cycle	After all cycles	Baseline	After one cycle	After all cycles	Baseline	After one cycle	After all cycles
Leukocytes	**II**	**II**	**I**	0	0	0	0	0	0
Hemoglobin	**I**	**I**	**I**	0	0	0	**I**	**I**	**I**
Platelets	**I**	**I**	**I**	0	0	0	0	0	0
Creatinine	0	0	0	0	0	0	0	0	0
Liver function	0	0	0	0	0	0	0	0	0
Creatinine	0	0	0	0	0	0	0	0	0
GFR	0	0	0	0	0	0	0	0	0

Significant changes are marked in bold

## Discussion

We report on the first three consecutive patients treated with a novel [^177^Lu]Lu-6A10-Fab conjugate. Previous studies focusing on ^90^Y- or ^131^I-labelled antibodies were mainly targeting tenascin or other tumor microenvironment targets in GBM [3, 10]. Only few antibodies against targets overexpressed directly on malignant glioma cells exist [11], such as CA-XII [12]. Targeting tumor cells directly improves effectiveness and reduces side effects. Moreover, ubiquitous expression of the target on the tumor cell, as previously reported for CA-XII [12, 13] is essential for a high therapeutic yield. High and long lasting radiochemical purity and specific binding with low expression in healthy brain are prerequisites of iRIT as highlighted in preclinical studies for [^177^Lu]Lu-6A10-Fab [4]. Because of the infiltrative growth of glioma, most target tumor cells are located distant but close to the resection cavity [1]. Using an antibody fragment instead of an antibody improves diffusion properties and allows for easier migration into the adjacent tissue [4]. In addition to radioimmunotherapy, several other intracavitary therapies have been performed with locally applied substances ranging from chemotherapeutics to radiolabeled small molecules targeting antigens in the tumor microenvironment and glioblastoma cells and novel CAR-T-cell therapy[14–18].

Lutetium-177 offers optimal theranostic properties with a shorter soft tissue range compared to Yttrium-90 and a gamma component well suited for scintigraphic imaging. It leads to better image quality and lower body radiation dose compared to Iodine-131 [4]. Also, dose estimation is more precise with Lutetium-177, especially when compared to Yttrium-90. While a therapeutic effect is expected to depend on the absorbed energy dose in tumor tissue, this absorbed dose is dependent on the relationship of injected activity and volume of the resection cavity [19]. Thus, defining the injected activity based on the volume of the resection is expected to be superior to standard fixed activity definitions [8, 19].

Our group has already elaborated on the landscape of iRIT studies [2, 5] and found that iRIT was mainly used for treatment of progressive disease or in combination with adjuvant therapies [6, 13, 20] with encouraging survival rates [2, 10]. Still, approved maintenance therapies for GBM are lacking and patients are essentially left without viable treatment options before inevitable occurrence of tumor progression. Our iRIT approach might fill this gap between standard therapy and treatment of progressive disease.

The frequency and kind of systemic side effects of iRIT are generally dependent on leakage into the blood and on absorbed doses in healthy brain tissue. Organs potentially affected are the kidneys [4, 21]; however, absorbed doses in our cohort were well below the known limits from external beam radiation [22]. Hematological abnormalities seen in our patients were lasting from previous radiochemotherapy regimens and not directly related to iRIT [23]. The absence of extracerebral side effects may be explained either by the only limited entry of the antibody fragment into the blood stream or by the only moderate activity injected into the resection cavity when compared to many systemic Lutetium-177-based radioligand therapies.

It is unclear at this stage, whether no therapy-related neurological side effects occurred since patient 2 developed seizures during therapy. However, confirmed tumor progression is a likely cause for the seizures. Results from the larger cohort of treated patients within the recruiting study are expected to better estimate the likelihood of neurological side effects. Reardon et al., applying a resection cavity volume dependent dosage of ^131^I-labelled tenascin C antibodies, report on no irreversible neurological side effects [24]. After applying fixed dosages resulting in a wider range of absorbed doses in adjacent tissue, other studies report on higher rates of side effects [2, 8]. Interestingly, we noted asymptomatic mild post-therapeutic contrast enhancement in patients 1 and 3, which can be regarded as a therapy-associated change.

The addition of dosimetry of radiation applied to the brain tissue around the resection cavity would be very interesting and is indeed an important topic of the prospective study. It is, however, not straight-forward and hampered especially by the steep activity gradient between activity within the cavity and neighboring tissue. We aim to establish reliable dosimetry models for the calculation of local (tumor-)dose when more patient data are available from the prospective phase I study.

## Conclusions

Intracavitary RIT with [^177^Lu]Lu-6A10-Fab as a novel therapeutic option for GBM patients appears to be safe and well tolerated albeit only assessed in three patients so far. The open multicenter phase I study (NCT05533242) is currently recruiting patients in three German brain tumor centers and will primarily evaluate the maximum tolerated dose and dose limiting toxicity in a 3 + 3 dose escalation design.

### Supplementary Information

**Additional file 1. Figures S1.** Post therapeutic whole body images of all patients from 2h p.i. to 7 days p.i.

## Data Availability

All data are included in the manuscript.

## Abbreviations

iRIT: Intracavitary radioimmunotherapy
GBM: Glioblastoma multiforme
Ca XII: Carboxyanhydrase XII
CTCAE: Common terminology criteria for adverse events
