# Supplementary figures and images for: First clinical experience with fractionated intracavitary radioimmunotherapy using [177Lu]Lu-6A10-Fab fragments in patients with glioblastoma: a pilot study
Source: EJNMMI Res. 2023 Sep 4;13:78. doi: 10.1186/s13550-023-01029-7 (PMC10477153; doi:10.1186/s13550-023-01029-7)

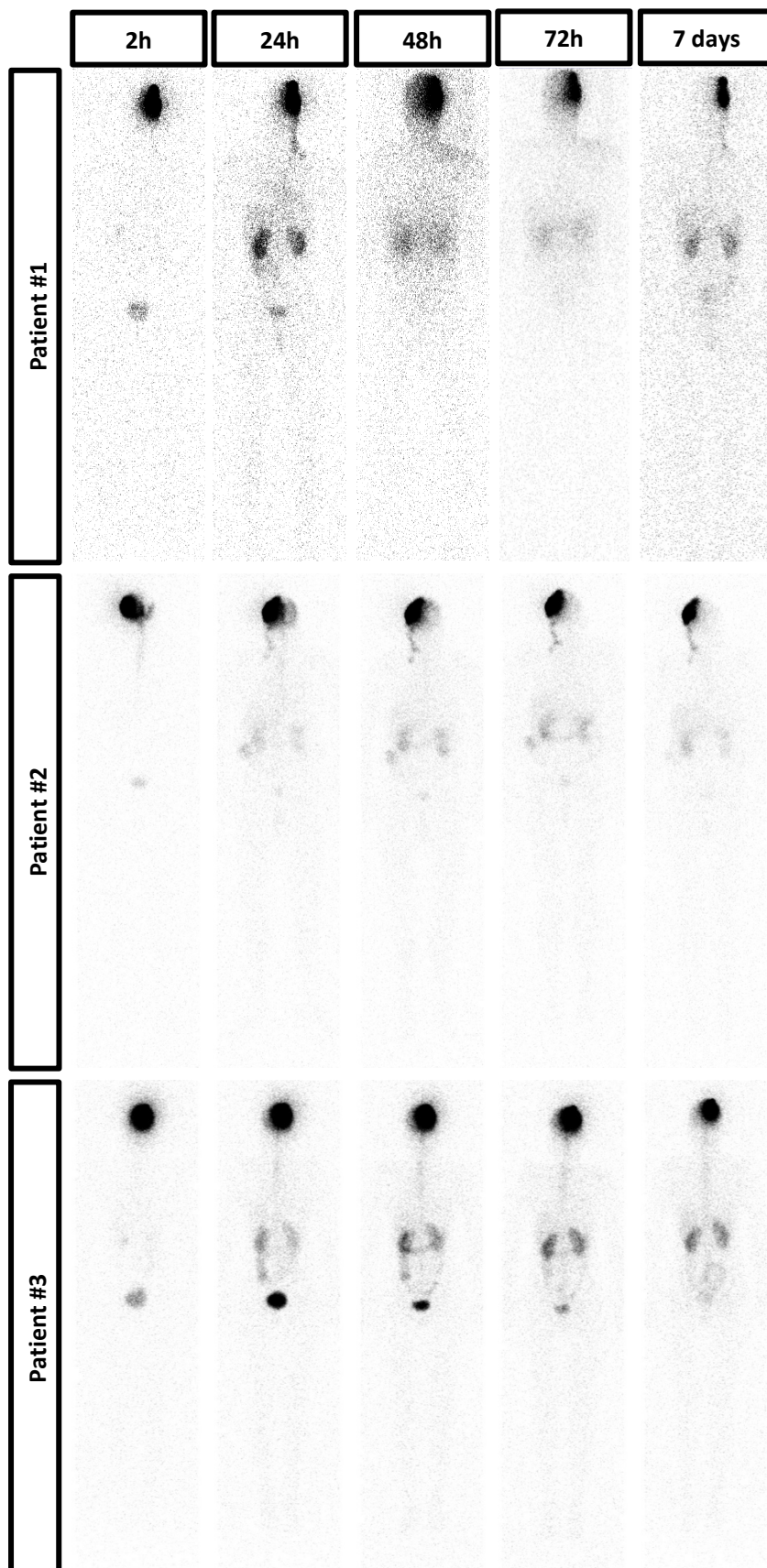

**Supplemental Figure 1:**

Post therapeutic whole body images of all patients from 2h p.i. to 7 days p.i..

Supplement: Supplementary file 1 — Additional file 1. Figures S1. Post therapeutic whole body images of all patients from 2h p.i. to 7 days p.i. [file 13550_2023_1029_MOESM1_ESM.pdf]
